# Cardiodynamic evaluation of sorfequiline (TBAJ-876): results from a first-in-human study

**DOI:** 10.1128/aac.01273-25

**Published:** 2026-01-30

**Authors:** Borje Darpo, Jerry Nedelman, Rebecca Bruning-Barry, Dean Hickman, Robert Kleiman, Antonio Lombardi, Hongqi Xue

**Affiliations:** 1 Clario 63912 , Philadelphia, Pennsylvania, USA; 2 TB Alliance 486654, New York, New York, USA; 3 RTI International 6856, Durham, North Carolina, USA; City St George's University of London, London, United Kingdom

**Keywords:** tuberculosis, QTc, concentration-QTc (C-QTc), multidrug resistance, first-in-human

## Abstract

Sorfequiline (TBAJ-876) is a novel diarylquinoline under development for tuberculosis. In a first-in-human, multiple-ascending-dose study, electrocardiogram and pharmacokinetic data were analyzed to assess cardiac repolarization. Placebo-corrected change-from-baseline in QTcF (ΔΔQTcF) ranged from −11.4 to +2.4 ms without dose dependency. Concentration–QTc modeling showed a shallow, non-significant slope. The predicted mean effect on ΔΔQTcF at the maximum geometric mean C_max_ of sorfequiline’s M3 metabolite was −1.1 ms (90% CI −9.5 to 7.4).

**CLINICAL TRIALS:**

This study is registered with ClinicalTrials.gov as NCT06058299.

## INTRODUCTION

Bedaquiline, a diarylquinoline approved for multidrug-resistant tuberculosis (1–4), prolongs the corrected QT (QTc) interval through its N-desmethyl metabolite (4–8). Because several tuberculosis drugs prolong QTc, the potential for additive effects remains a safety concern (6, 7, 9–14). Sorfequiline (TBAJ-876) is a next-generation diarylquinoline discovered by the TB Alliance to maintain potent antimycobacterial activity while minimizing risk of QTc prolongation (15). Sorfequiline also has a major, active, N-desmethyl metabolite, called M3, and a minor, less active, N,N-didesmethyl metabolite, called M2. The present analysis summarizes results from the multiple-ascending-dose (MAD) part of the first-in-human (FIH) study (CL-001) (15) evaluating the effects of sorfequiline on cardiac conduction and QTc. Both time-point and concentration–QTc analyses were conducted to characterize potential proarrhythmic risk (16, 17).

Healthy adults aged 19–50 years were enrolled in a randomized, placebo-controlled, MAD study of sorfequiline suspension (ClinicalTrials.gov identifier NCT06058299) (15). Subjects received once-daily oral doses of 25, 75, or 200 mg for 14 days under fed conditions. Across the three groups, 26 participants received sorfequiline and 12 received placebo.

Continuous electrocardiogram (ECG) recordings were obtained via Holter monitoring on Days 1 and 14 and extracted at prespecified postdose time points (0.5–24 h). QT intervals were corrected for heart rate using Fridericia’s formula (QTcF) (16). The primary endpoint was change-from-baseline QTcF (ΔQTcF), with placebo-corrected ΔQTcF (ΔΔQTcF) as the principal summary. Additional parameters (heart rate, PR, QRS) were evaluated using the same by-time-point model as for QTcF.

Plasma concentrations of sorfequiline and its metabolites (M2, M3) were determined from matched pharmacokinetic samples. Linear mixed-effects modeling was used to quantify the relationship between ΔQTcF and analyte concentrations (C-QTc analysis) (18). Competing models were compared by the Akaike information criterion, and parameter estimates were obtained using restricted maximum likelihood. A clinically meaningful QTc prolongation was predefined as an effect on ΔΔQTcF ≥ 10 ms at clinically relevant plasma levels, as shown by the upper bound of the two-sided 90% confidence interval (CI) of the model-estimated QTc effect (∆∆QTcF) at the observed geometric mean (GM) C_max_ (18); a clinically meaningful effect on QTc would be excluded if the upper 90% limit for ∆∆QTcF was below 10 ms.

Thirty-eight subjects were included in the analysis (9 each at 25 mg and 75 mg, 8 at 200 mg, and 12 placebo). One subject in the 200 mg group was excluded for missing baseline ECGs. Baseline ECG measures were within normal ranges for all participants included in the analysis (mean QTcF 397–409 ms; heart rate 58–78 bpm).

**Fig 1:**
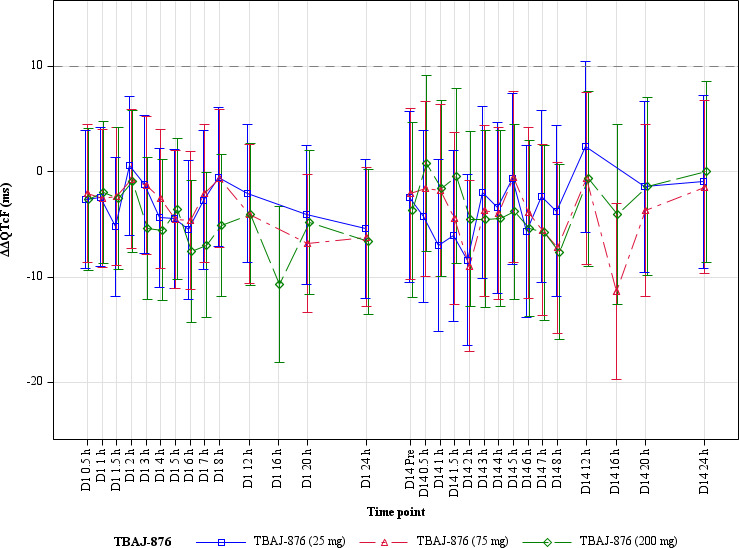
Placebo-corrected change-from-baseline QTcF (ΔΔQTcF), and 90% CIs, across dose groups and time points.

There was no clinically relevant effect on heart rate, as the mean placebo-corrected heart rate change was < 10 bpm at all time points, nor on cardiac conduction, i.e, the PR and QRS intervals. Mean ΔΔQTcF values showed no evidence of QTc prolongation at any dose (Fig. 1). On Day 1, ΔΔQTcF ranged from −10.7 to −1.0 ms across time points in the 200 mg group; on Day 14, from −7.6 to +0.8 ms. The upper bound of the two-sided 90% CI exceeded +10 ms at only one time point (12 h post-dose, 25 mg group: 2.4 ms; 90% CI −5.8 to 10.5), with no dose- or time-related pattern.

**Fig 2:**
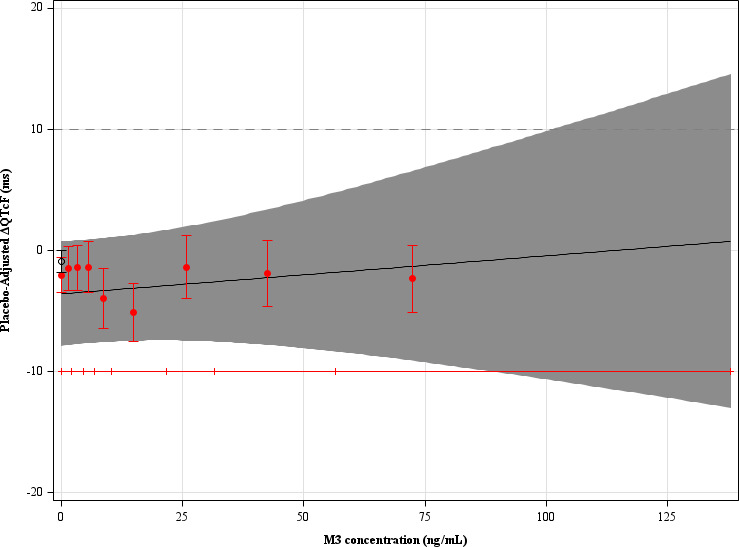
Model-predicted ΔΔQTcF (mean as black line, 90% CIs as shaded region) and estimated mean ΔΔQTcF by decile of M3 (red circles with vertical bars).

C-QTc analysis included 850 matched concentration-ΔQTcF pairs from the active and placebo treatment groups. Plasma concentrations of sorfequiline and M3 were modestly correlated. M2 and M3 were highly correlated (r = 0.99); therefore, models including both metabolites were excluded. Among the tested models, the one using M3 (the major circulating metabolite and analogous metabolite to bedaquiline’s M2) as the single analyte provided the best fit. Figure 2 shows the goodness-of-fit of the model.

The slope of the relationship between M3 concentration and ΔQTcF was shallow and statistically nonsignificant at the 10% significance level (0.031 ms per ng/mL; 90% CI −0.068 to 0.13), with a treatment effect-specific intercept of −3.6 ms (90% CI −7.9 to 0.74). The predicted ΔΔQTcF at the GM C_max_ of M3 (79.7 ng/mL) on Day 14 in the 200 mg group was −1.1 ms (90% CI −9.5 to 7.4). For the model using parent drug concentrations, the slope was 0.00053 ms per ng/mL (90% CI −0.0051 to 0.0062), with a treatment effect-specific intercept of −2.0 ms (90% CI −6.1 to 2.1). The predicted effect on ΔΔQTcF at the GM C_max_ of sorfequiline (1,143 ng/mL) on Day 14 in the 200 mg group was -1.4 ms (90% CI 9.1 to 6.3). An effect on ΔΔQTcF exceeding 10 ms can be excluded up to the observed ranges of M3 and sorfequiline plasma concentration of ~102 and ~1800 ng/mL, respectively.

Estimated parameters from the fitted models are provided as Supplementary Material.

No participant exhibited absolute QTcF > 480 ms or an increase > 60 ms from baseline. Findings were consistent between by-time-point and C-QTc analyses, supporting the absence of a clinically relevant QTc effect of sorfequiline following 14 days of dosing up to 200 mg once daily.

In this FIH study, sorfequiline produced no clinically meaningful effect on cardiac repolarization, heart rate, or other ECG parameters in healthy volunteers. These findings corroborate nonclinical evaluations, in which sorfequiline and its metabolites showed low potential for hERG inhibition and did not impact ECG parameters, including QTc prolongation, in a 12-day cardiovascular safety pharmacology study, nor in 13- and 26-week toxicology studies in dogs at exposures of M3 and sorfequiline up to 10-fold and 2-fold higher, respectively, than mean C_max_ values on Day 14 for the 200 mg dose in this study.

While the present study was limited by short duration and enrollment of healthy subjects, the results provide early clinical evidence of a low risk for QTc prolongation. Continued evaluation in patients with tuberculosis will be important to confirm cardiac safety during multidrug therapy, where potential pharmacokinetic interactions and comorbidities may alter exposure, and regimen partners may affect QTc. Together, the data support further clinical development of sorfequiline as a component of investigational tuberculosis regimens.

## SUPPLEMENTAL MATERIAL

Tables S1 to S5 aac.01273-25-s0001.docx Model parameter estimates.

ASM does not own the copyrights to Supplemental Material that may be linked to, or accessed through, an article. The authors have granted ASM a non-exclusive, world-wide license to publish the Supplemental Material files. Please contact the corresponding author directly for reuse.

## References

[1] Martín-García M, Esteban J. 2021. Evaluating bedaquiline as a treatment option for multidrug-resistant tuberculosis. Expert Opin Pharmacother 22:535–541. doi:10.1080/14656566.2020.1867538 33393406

[2] Singh B, Singh C. 2023. Bedaquiline in drug-resistant tuberculosis: a mini-review. Curr Mol Pharmacol 16:243–253. doi:10.2174/1874467215666220421130707 36919348

[3] Guglielmetti L, Chiesi S, Eimer J, Dominguez J, Masini T, Varaine F, Veziris N, Ader F, Robert J. 2020. Bedaquiline and delamanid for drug-resistant tuberculosis: a clinician’s perspective. Future Microbiol 15:779–799. doi:10.2217/fmb-2019-0309 32700565

[4] FDA. 2024. Bedaquiline US label. Available from: https://www.accessdata.fda.gov/drugsatfda_docs/label/2024/204384s019lbl.pdf

[5] Darmayani IGAAPS, Ascobat P, Instiaty I, Sugiri YJR, Sawitri N. 2022. Bedaquiline effect on QT interval of drugs-resistant tuberculosis patients: real world data. Acta Med Indones 54:389–396. 36156479

[6] Dooley KE, Rosenkranz SL, Conradie F, Moran L, Hafner R, von Groote-Bidlingmaier F, Lama JR, Shenje J, De Los Rios J, Comins K, et al.. 2021. QT effects of bedaquiline, delamanid, or both in patients with rifampicin-resistant tuberculosis: a phase 2, open-label, randomised, controlled trial. Lancet Infect Dis 21:975–983. doi:10.1016/S1473-3099(20)30770-2 33587897 PMC8312310

[7] Putra ON, Yulistiani Y, Soedarsono S. 2022. Scoping review: QT interval prolongation in regimen containing bedaquiline and delamanid in patients with drug-resistant tuberculosis. Int J Mycobacteriol 11:349–355. doi:10.4103/ijmy.ijmy_178_22 36510917

[8] FDA. 2012. Bedaquiline other reviews. Available from: https://www.accessdata.fda.gov/drugsatfda_docs/nda/2012/204384Orig1s000OtherR.pdf

[9] FDA. 2024 FDA pretomanid label. Available from: https://www.accessdata.fda.gov/drugsatfda_docs/label/2024/212862s008lbl.pdf

[10] Abdelwahab MT, Court R, Everitt D, Diacon AH, Dawson R, Svensson EM, Maartens G, Denti P. 2021. Effect of clofazimine concentration on QT prolongation in patients treated for tuberculosis. Antimicrob Agents Chemother 65:e0268720. doi:10.1128/AAC.02687-20 33875426 PMC8218646

[11] Esposito S, Bianchini S, Blasi F. 2015. Bedaquiline and delamanid in tuberculosis. Expert Opin Pharmacother 16:2319–2330. doi:10.1517/14656566.2015.1080240 26293803

[12] Gupta R, Geiter LJ, Hafkin J, Wells CD. 2015. Delamanid and QT prolongation in the treatment of multidrug-resistant tuberculosis. Int J Tuberc Lung Dis 19:1261–1262. doi:10.5588/ijtld.15.0541 26459547

[13] Yun HY, Chang V, Radtke KK, Wang Q, Strydom N, Chang MJ, Savic RM. 2022. Model-based efficacy and toxicity comparisons of moxifloxacin for multidrug-resistant tuberculosis. Open Forum Infect Dis 9:ofab660. doi:10.1093/ofid/ofab660 35146045 PMC8825669

[14] Li H, Salinger DH, Everitt D, Li M, Del Parigi A, Mendel C, Nedelman JR. 2019. Long-term effects on QT prolongation of pretomanid alone and in combinations in patients with tuberculosis. Antimicrob Agents Chemother 63:e00445-19. doi:10.1128/AAC.00445-19 31358590 PMC6761551

[15] Lombardi A, Pappas F, Nedelman J, Hickman D, Jaw-Tsai S, Olugbosi M, Bruinenberg P, Beumont M, Sun E. 2024. Pharmacokinetics and safety of TBAJ-876, a novel antimycobacterial diarylquinoline, in healthy subjects. Antimicrob Agents Chemother 68:e0061324. doi:10.1128/aac.00613-24 39194204 PMC11460996

[16] Darpo B, Fossa AA, Couderc JP, Zhou M, Schreyer A, Ticktin M, Zapesochny A. 2011. Improving the precision of QT measurements. Cardiol J 18:401–410. 21769821

[17] Garnett CE, Beasley N, Bhattaram VA, Jadhav PR, Madabushi R, Stockbridge N, Tornøe CW, Wang Y, Zhu H, Gobburu JV. 2008. Concentration-QT relationships play a key role in the evaluation of proarrhythmic risk during regulatory review. J Clin Pharmacol 48:13–18. doi:10.1177/0091270007307881 18094216

[18] Garnett C, Bonate PL, Dang Q, Ferber G, Huang D, Liu J, Mehrotra D, Riley S, Sager P, Tornoe C, Wang Y. 2018. Scientific white paper on concentration-QTc modeling. J Pharmacokinet Pharmacodyn 45:383–397. doi:10.1007/s10928-017-9558-5 29209907

