# Supplementary material for: Cardiodynamic evaluation of sorfequiline (TBAJ-876): results from a first-in-human study
Source: Antimicrob Agents Chemother. 2026 Jan 30;70(3):e01273-25. doi: 10.1128/aac.01273-25 (PMC12959150; doi:10.1128/aac.01273-25)
Supplement: Tables S1 to S5 — Model parameter estimates. [file aac.01273-25-s0001.docx]

# Supplementary Material for:

# Cardiodynamic Evaluation of Sorfequiline (TBAJ-876): Results from a First-in-Human Study

Borje Darpo, MD, PhD¹, Jerry Nedelman, PhD², Rebecca Bruning-Barry, PhD³, Dean Hickman, DPhil², Robert Kleiman, MD¹, Antonio Lombardi, MD², Hongqi Xue, PhD¹

¹ Clario, Philadelphia, USA
² TB Alliance, New York, NY, USA
³ RTI International, Durham, NC, USA

Corresponding author:
Jerry Nedelman, PhD
TB Alliance
80 Pine Street, 20th Floor
New York, NY 10005
Email: jerry.nedelman@tballiance.org

Table S1 Concentration-QTc analysis of TBAJ-876 and M2 and associated ΔQTcF prolongation for Model A (Concentration-QTc analysis set)

| **Parameter** | **Estimate** | **SE** | ***df*** | ***t*-Value** | ***P*Value** | **90% CI** |
| --- | --- | --- | --- | --- | --- | --- |
| Treatment Effect (ms) | -2.461 | 2.3562 | 34.4 | -1.04 | 0.3036 | -6.4439, 1.5220 |
| TBAJ-876 Slope (ms per ng/mL) | 0.00084 | 0.0037 | 8.9 | 0.22 | 0.8275 | -0.006031, 0.007711 |
| M2 Slope (ms per ng/mL) | 0.045 | 0.3044 | 20.3 | 0.15 | 0.8852 | -0.4801, 0.5692 |
| Day 1 0.5 h Post-dose Effect (ms) | 1.98 | 2.1370 | 51.5 | 0.93 | 0.3577 | -1.596, 5.563 |
| Day 1 1 h Post-dose Effect (ms) | 0.93 | 2.1292 | 50.8 | 0.44 | 0.6642 | -2.638, 4.497 |
| Day 1 2 h Post-dose Effect (ms) | -3.769 | 2.1300 | 50.5 | -1.77 | 0.0828 | -7.3384, -0.2004 |
| Day 1 3 h Post-dose Effect (ms) | -4.948 | 2.1379 | 51.1 | -2.31 | 0.0247 | -8.5297, -1.3667 |
| Day 1 4 h Post-dose Effect (ms) | -3.059 | 2.1396 | 51.3 | -1.43 | 0.1589 | -6.6430, 0.5251 |
| Day 1 5 h Post-dose Effect (ms) | -0.0753 | 2.1438 | 51.5 | -0.04 | 0.9721 | -3.66611, 3.51559 |
| Day 1 6 h Post-dose Effect (ms) | 2.35 | 2.1351 | 50.8 | 1.10 | 0.2766 | -1.229, 5.926 |
| Day 1 8 h Post-dose Effect (ms) | 0.91 | 2.1220 | 50.0 | 0.43 | 0.6694 | -2.645, 4.468 |
| Day 1 12 h Post-dose Effect (ms) | 2.55 | 2.1201 | 50.0 | 1.20 | 0.2347 | -1.003, 6.103 |
| Day 1 16 h Post-dose Effect (ms) | 10.25 | 2.5365 | 99.3 | 4.04 | 0.0001 | 6.042, 14.464 |
| Day 1 20 h Post-dose Effect (ms) | 6.17 | 2.1284 | 50.8 | 2.90 | 0.0055 | 2.607, 9.739 |
| Day 1 24 h Post-dose Effect (ms) | -0.736 | 2.1402 | 51.9 | -0.34 | 0.7324 | -4.3202, 2.8484 |
| Day 14 Pre-dose Effect (ms) | 1.22 | 2.1386 | 51.7 | 0.57 | 0.5716 | -2.364, 4.799 |
| Day 14 0.5 h Post-dose Effect (ms) | -1.353 | 2.1512 | 52.8 | -0.63 | 0.5320 | -4.9548, 2.2483 |
| Day 14 1 h Post-dose Effect (ms) | -0.968 | 2.1335 | 51.1 | -0.45 | 0.6520 | -4.5418, 2.6064 |
| Day 14 2 h Post-dose Effect (ms) | -4.130 | 2.1423 | 51.4 | -1.93 | 0.0594 | -7.7180, -0.5413 |
| Day 14 3 h Post-dose Effect (ms) | -4.832 | 2.1604 | 52.9 | -2.24 | 0.0296 | -8.4486, -1.2148 |
| Day 14 4 h Post-dose Effect (ms) | -2.331 | 2.1645 | 53.2 | -1.08 | 0.2864 | -5.9543, 1.2924 |
| Day 14 5 h Post-dose Effect (ms) | 1.51 | 2.1760 | 53.8 | 0.69 | 0.4921 | -2.137, 5.147 |
| Day 14 8 h Post-dose Effect (ms) | 0.69 | 2.1338 | 51.1 | 0.32 | 0.7476 | -2.884, 4.265 |
| Day 14 12 h Post-dose Effect (ms) | 1.90 | 2.1374 | 51.5 | 0.89 | 0.3787 | -1.682, 5.478 |
| Day 14 16 h Post-dose Effect (ms) | 2.89 | 2.2588 | 64.1 | 1.28 | 0.2057 | -0.882, 6.658 |
| Day 14 20 h Post-dose Effect (ms) | 5.94 | 2.1444 | 52.2 | 2.77 | 0.0077 | 2.353, 9.534 |
| Day 14 24 h Post-dose Effect (ms) | 0.69 | 2.1605 | 53.8 | 0.32 | 0.7491 | -2.921, 4.311 |
| Centered Baseline Effect (ms) | -0.0795 | 0.0579 | 21.5 | -1.37 | 0.1836 | -0.17904, 0.01998 |

Based on a linear mixed-effects model with ΔQTcF as the dependent variable, time-matched TBAJ-876, and M2 plasma concentration as an explanatory variable, centered baseline QTcF as an additional covariate, treatment (active = 1 or placebo = 0) and time as fixed effects, and a random intercept and slopes per subject. Fitted by REML estimation.

Table S2 Concentration-QTc analysis of TBAJ-876 and M3 and associated ΔQTcF prolongation for Model B (Concentration-QTc analysis set)

| **Parameter** | **Estimate** | **SE** | ***df*** | ***t*-Value** | ***P*Value** | **90% CI** |
| --- | --- | --- | --- | --- | --- | --- |
| Treatment Effect (ms) | -2.513 | 2.3540 | 34.8 | -1.07 | 0.2931 | -6.4906, 1.4651 |
| TBAJ-876 Slope (ms per ng/mL) | 0.00022 | 0.0038 | 9.2 | 0.06 | 0.9550 | -0.006685, 0.007124 |
| M3 Slope (ms per ng/mL) | 0.027 | 0.0613 | 20.6 | 0.43 | 0.6685 | -0.0789, 0.1321 |
| Day 1 0.5 h Post-dose Effect (ms) | 2.01 | 2.1339 | 52.0 | 0.94 | 0.3502 | -1.562, 5.585 |
| Day 1 1 h Post-dose Effect (ms) | 0.98 | 2.1273 | 51.5 | 0.46 | 0.6482 | -2.587, 4.540 |
| Day 1 2 h Post-dose Effect (ms) | -3.705 | 2.1294 | 51.3 | -1.74 | 0.0879 | -7.2716, -0.1379 |
| Day 1 3 h Post-dose Effect (ms) | -4.870 | 2.1359 | 51.8 | -2.28 | 0.0268 | -8.4472, -1.2927 |
| Day 1 4 h Post-dose Effect (ms) | -2.997 | 2.1365 | 51.9 | -1.40 | 0.1666 | -6.5751, 0.5811 |
| Day 1 5 h Post-dose Effect (ms) | -0.0108 | 2.1394 | 52.0 | -0.01 | 0.9960 | -3.59362, 3.57206 |
| Day 1 6 h Post-dose Effect (ms) | 2.41 | 2.1292 | 51.2 | 1.13 | 0.2636 | -1.160, 5.973 |
| Day 1 8 h Post-dose Effect (ms) | 0.92 | 2.1154 | 50.3 | 0.44 | 0.6644 | -2.621, 4.468 |
| Day 1 12 h Post-dose Effect (ms) | 2.58 | 2.1131 | 50.2 | 1.22 | 0.2278 | -0.961, 6.121 |
| Day 1 16 h Post-dose Effect (ms) | 10.23 | 2.5286 | 99.8 | 4.05 | 0.0001 | 6.031, 14.428 |
| Day 1 20 h Post-dose Effect (ms) | 6.17 | 2.1219 | 51.0 | 2.91 | 0.0054 | 2.611, 9.720 |
| Day 1 24 h Post-dose Effect (ms) | -0.762 | 2.1330 | 52.1 | -0.36 | 0.7222 | -4.3344, 2.8095 |
| Day 14 Pre-dose Effect (ms) | 1.21 | 2.1336 | 52.1 | 0.57 | 0.5720 | -2.360, 4.786 |
| Day 14 0.5 h Post-dose Effect (ms) | -1.296 | 2.1463 | 53.2 | -0.60 | 0.5485 | -4.8889, 2.2967 |
| Day 14 1 h Post-dose Effect (ms) | -0.960 | 2.1273 | 51.4 | -0.45 | 0.6537 | -4.5235, 2.6033 |
| Day 14 2 h Post-dose Effect (ms) | -4.045 | 2.1374 | 51.8 | -1.89 | 0.0640 | -7.6244, -0.4650 |
| Day 14 3 h Post-dose Effect (ms) | -4.761 | 2.1549 | 53.3 | -2.21 | 0.0315 | -8.3685, -1.1542 |
| Day 14 4 h Post-dose Effect (ms) | -2.274 | 2.1600 | 53.6 | -1.05 | 0.2971 | -5.8897, 1.3410 |
| Day 14 5 h Post-dose Effect (ms) | 1.59 | 2.1729 | 54.4 | 0.73 | 0.4684 | -2.049, 5.223 |
| Day 14 8 h Post-dose Effect (ms) | 0.75 | 2.1296 | 51.6 | 0.35 | 0.7268 | -2.819, 4.315 |
| Day 14 12 h Post-dose Effect (ms) | 1.92 | 2.1338 | 52.0 | 0.90 | 0.3725 | -1.654, 5.493 |
| Day 14 16 h Post-dose Effect (ms) | 2.93 | 2.2538 | 64.6 | 1.30 | 0.1977 | -0.827, 6.695 |
| Day 14 20 h Post-dose Effect (ms) | 5.95 | 2.1403 | 52.7 | 2.78 | 0.0075 | 2.365, 9.532 |
| Day 14 24 h Post-dose Effect (ms) | 0.68 | 2.1566 | 54.2 | 0.31 | 0.7552 | -2.933, 4.285 |
| Centered Baseline Effect (ms) | -0.0850 | 0.0581 | 22.7 | -1.46 | 0.1573 | -0.18457, 0.01465 |

Based on a linear mixed-effects model with ΔQTcF as the dependent variable, time-matched TBAJ-876, and M3 plasma concentration as an explanatory variable, centered baseline QTcF as an additional covariate, treatment (active = 1 or placebo = 0) and time as fixed effects, and a random intercept and slopes per subject. Fitted by REML estimation.

Table S3 Concentration-QTc analysis of TBAJ-876 and associated ΔQTcF prolongation for Model C (Concentration-QTc analysis set)

| **Parameter** | **Estimate** | **SE** | ***df*** | ***t-*Value** | **𝑃-Value** | **90% CI** |
| --- | --- | --- | --- | --- | --- | --- |
| Treatment Effect (ms) | −2.01 | 2.4157 | 33.8 | -0.83 | 0.4107 | −6.097, 2.073 |
| TBAJ-876 Slope (ms per ng/mL) | 0.00053 | 0.0032 | 11.3 | 0.17 | 0.8695 | −0.005144, 0.006209 |
| Centered Baseline Effect (ms) | −0.080 | 0.0573 | 21.2 | -1.39 | 0.1790 | −0.1782, 0.0189 |
| Day 1 0.5 h Post-dose Effect (ms) | 1.73 | 2.2144 | 52.6 | 0.78 | 0.4386 | −1.979, 5.436 |
| Day 1 1 h Post-dose Effect (ms) | 0.72 | 2.2031 | 51.6 | 0.33 | 0.7461 | −2.973, 4.407 |
| Day 1 2 h Post-dose Effect (ms) | −3.97 | 2.1938 | 50.5 | -1.81 | 0.0764 | −7.644, −0.292 |
| Day 1 3 h Post-dose Effect (ms) | −5.15 | 2.1966 | 50.7 | -2.35 | 0.0229 | −8.833, −1.472 |
| Day 1 4 h Post-dose Effect (ms) | −3.40 | 2.1973 | 50.8 | -1.55 | 0.1275 | −7.086, 0.277 |
| Day 1 5 h Post-dose Effect (ms) | −0.49 | 2.2004 | 51.0 | -0.22 | 0.8252 | −4.175, 3.198 |
| Day 1 6 h Post-dose Effect (ms) | 1.97 | 2.1951 | 50.6 | 0.90 | 0.3740 | −1.709, 5.647 |
| Day 1 8 h Post-dose Effect (ms) | 0.58 | 2.1902 | 50.4 | 0.26 | 0.7927 | −3.091, 4.249 |
| Day 1 12 h Post-dose Effect (ms) | 2.31 | 2.1935 | 50.8 | 1.05 | 0.2978 | −1.367, 5.983 |
| Day 1 16 h Post-dose Effect (ms) | 9.79 | 2.6499 | 104.8 | 3.69 | 0.0004 | 5.391, 14.187 |
| Day 1 20 h Post-dose Effect (ms) | 5.94 | 2.2052 | 51.9 | 2.69 | 0.0095 | 2.247, 9.634 |
| Day 1 24 h Post-dose Effect (ms) | −1.06 | 2.2180 | 53.0 | -0.48 | 0.6349 | −4.772, 2.654 |
| Day 14 Pre-dose Effect (ms) | 1.46 | 2.2044 | 51.7 | 0.66 | 0.5107 | −2.232, 5.152 |
| Day 14 0.5 h Post-dose Effect (ms) | −0.79 | 2.2165 | 52.8 | -0.36 | 0.7220 | −4.504, 2.918 |
| Day 14 1 h Post-dose Effect (ms) | −0.71 | 2.2018 | 51.4 | -0.32 | 0.7490 | −4.396, 2.980 |
| Day 14 2 h Post-dose Effect (ms) | −3.83 | 2.2151 | 52.3 | -1.73 | 0.0901 | −7.535, −0.116 |
| Day 14 3 h Post-dose Effect (ms) | −4.38 | 2.2311 | 53.7 | -1.96 | 0.0546 | −8.118, −0.649 |
| Day 14 4 h Post-dose Effect (ms) | −2.03 | 2.2356 | 54.0 | -0.91 | 0.3689 | −5.767, 1.716 |
| Day 14 5 h Post-dose Effect (ms) | 1.80 | 2.2457 | 54.6 | 0.80 | 0.4251 | −1.953, 5.562 |
| Day 14 8 h Post-dose Effect (ms) | 0.96 | 2.2017 | 51.4 | 0.44 | 0.6645 | −2.728, 4.648 |
| Day 14 12 h Post-dose Effect (ms) | 2.18 | 2.2010 | 51.3 | 0.99 | 0.3262 | −1.505, 5.869 |
| Day 14 16 h Post-dose Effect (ms) | 3.42 | 2.3239 | 63.6 | 1.47 | 0.1463 | −0.461, 7.297 |
| Day 14 20 h Post-dose Effect (ms) | 6.28 | 2.2105 | 52.3 | 2.84 | 0.0064 | 2.578, 9.981 |
| Day 14 24 h Post-dose Effect (ms) | 1.03 | 2.2274 | 53.9 | 0.46 | 0.6462 | −2.700, 4.756 |

Based on a linear mixed-effects model with ΔQTcF as the dependent variable, time-matched TBAJ-876 plasma concentration as an explanatory variable, centered baseline QTcF as an additional covariate, treatment (active = 1 or placebo = 0) and time as fixed effects, and a random intercept and random slope per subject. Fitted by REML estimation.

Table S4 Concentration-QTc analysis of M2 and associated ΔQTcF prolongation for Model D (Concentration-QTc analysis set)

| **Parameter** | **Estimate** | **SE** | ***df*** | ***t-*Value** | **𝑃-Value** | **90% CI** |
| --- | --- | --- | --- | --- | --- | --- |
| Treatment Effect (ms) | −3.52 | 2.5454 | 35.2 | -1.38 | 0.1751 | −7.823, 0.777 |
| M2 Slope (ms per ng/mL) | 0.11 | 0.2822 | 18.1 | 0.38 | 0.7074 | −0.382, 0.597 |
| Centered Baseline Effect (ms) | −0.18 | 0.0601 | 35.4 | -2.98 | 0.0052 | −0.280, −0.078 |
| Day 1 0.5 h Post-dose Effect (ms) | 2.72 | 2.2963 | 51.6 | 1.18 | 0.2419 | −1.128, 6.564 |
| Day 1 1 h Post-dose Effect (ms) | 1.72 | 2.2963 | 51.6 | 0.75 | 0.4565 | −2.123, 5.569 |
| Day 1 2 h Post-dose Effect (ms) | −2.89 | 2.2907 | 51.1 | -1.26 | 0.2128 | −6.728, 0.947 |
| Day 1 3 h Post-dose Effect (ms) | −4.10 | 2.2907 | 51.1 | -1.79 | 0.0797 | −7.934, −0.259 |
| Day 1 4 h Post-dose Effect (ms) | −2.47 | 2.2905 | 51.0 | -1.08 | 0.2855 | −6.309, 1.365 |
| Day 1 5 h Post-dose Effect (ms) | 0.34 | 2.2896 | 51.0 | 0.15 | 0.8829 | −3.497, 4.175 |
| Day 1 6 h Post-dose Effect (ms) | 2.93 | 2.2896 | 51.0 | 1.28 | 0.2058 | −0.902, 6.770 |
| Day 1 8 h Post-dose Effect (ms) | 1.64 | 2.2901 | 51.0 | 0.72 | 0.4778 | −2.199, 5.474 |
| Day 1 12 h Post-dose Effect (ms) | 3.27 | 2.2893 | 50.9 | 1.43 | 0.1587 | −0.561, 7.110 |
| Day 1 16 h Post-dose Effect (ms) | 11.26 | 2.6879 | 94.9 | 4.19 | < 0.0001 | 6.800, 15.730 |
| Day 1 20 h Post-dose Effect (ms) | 6.77 | 2.2942 | 51.4 | 2.95 | 0.0047 | 2.930, 10.616 |
| Day 1 24 h Post-dose Effect (ms) | −0.058 | 2.3041 | 52.2 | -0.03 | 0.9800 | −3.9164, 3.8003 |
| Day 14 Pre-dose Effect (ms) | 1.88 | 2.2956 | 51.5 | 0.82 | 0.4174 | −1.968, 5.722 |
| Day 14 0.5 h Post-dose Effect (ms) | −0.57 | 2.3106 | 52.8 | -0.25 | 0.8057 | −4.440, 3.297 |
| Day 14 1 h Post-dose Effect (ms) | −0.28 | 2.3004 | 51.9 | -0.12 | 0.9041 | −4.131, 3.574 |
| Day 14 2 h Post-dose Effect (ms) | −3.34 | 2.3017 | 52.0 | -1.45 | 0.1529 | −7.193, 0.516 |
| Day 14 3 h Post-dose Effect (ms) | −4.00 | 2.3138 | 53.0 | -1.73 | 0.0898 | −7.871, −0.124 |
| Day 14 4 h Post-dose Effect (ms) | −1.74 | 2.3137 | 53.1 | -0.75 | 0.4556 | −5.612, 2.134 |
| Day 14 5 h Post-dose Effect (ms) | 2.15 | 2.3125 | 52.9 | 0.93 | 0.3578 | −1.726, 6.017 |
| Day 14 8 h Post-dose Effect (ms) | 1.16 | 2.3022 | 52.0 | 0.50 | 0.6175 | −2.699, 5.012 |
| Day 14 12 h Post-dose Effect (ms) | 2.46 | 2.3031 | 52.1 | 1.07 | 0.2909 | −1.400, 6.314 |
| Day 14 16 h Post-dose Effect (ms) | 3.51 | 2.4106 | 62.4 | 1.46 | 0.1502 | −0.513, 7.537 |
| Day 14 20 h Post-dose Effect (ms) | 6.64 | 2.3011 | 52.0 | 2.89 | 0.0057 | 2.787, 10.494 |
| Day 14 24 h Post-dose Effect (ms) | 1.31 | 2.3163 | 53.3 | 0.56 | 0.5745 | −2.569, 5.186 |

Based on a linear mixed-effects model with ΔQTcF as the dependent variable, time-matched M2 plasma concentration as an explanatory variable, centered baseline QTcF as an additional covariate, treatment (active = 1 or placebo = 0) and time as fixed effects, and a random intercept and random slope per subject. Fitted by REML estimation.

Table S5 Concentration-QTc analysis of M3 and associated ΔQTcF prolongation for Model E (Concentration-QTc analysis set)

| **Parameter** | **Estimate** | **SE** | ***df*** | ***t-*Value** | **𝑃-Value** | **90% CI** |
| --- | --- | --- | --- | --- | --- | --- |
| Treatment Effect (ms) | −3.58 | 2.5574 | 35.3 | -1.40 | 0.1704 | −7.898, 0.741 |
| M3 Slope (ms per ng/mL) | 0.031 | 0.0571 | 18.1 | 0.55 | 0.5889 | −0.0675, 0.1304 |
| Centered Baseline Effect (ms) | −0.18 | 0.0603 | 35.4 | -3.00 | 0.0049 | −0.283, −0.079 |
| Day 1 0.5 h Post-dose Effect (ms) | 2.74 | 2.3029 | 51.4 | 1.19 | 0.2393 | −1.116, 6.599 |
| Day 1 1 h Post-dose Effect (ms) | 1.75 | 2.3029 | 51.4 | 0.76 | 0.4518 | −2.111, 5.603 |
| Day 1 2 h Post-dose Effect (ms) | −2.88 | 2.2969 | 50.9 | -1.25 | 0.2162 | −6.724, 0.972 |
| Day 1 3 h Post-dose Effect (ms) | −4.08 | 2.2955 | 50.8 | -1.78 | 0.0817 | −7.923, −0.231 |
| Day 1 4 h Post-dose Effect (ms) | −2.47 | 2.2944 | 50.7 | -1.08 | 0.2870 | −6.313, 1.375 |
| Day 1 5 h Post-dose Effect (ms) | 0.33 | 2.2924 | 50.5 | 0.14 | 0.8856 | −3.510, 4.173 |
| Day 1 6 h Post-dose Effect (ms) | 2.92 | 2.2917 | 50.5 | 1.27 | 0.2092 | −0.925, 6.755 |
| Day 1 8 h Post-dose Effect (ms) | 1.61 | 2.2919 | 50.5 | 0.70 | 0.4859 | −2.231, 5.449 |
| Day 1 12 h Post-dose Effect (ms) | 3.27 | 2.2907 | 50.4 | 1.43 | 0.1597 | −0.569, 7.108 |
| Day 1 16 h Post-dose Effect (ms) | 11.19 | 2.6871 | 93.6 | 4.16 | < 0.0001 | 6.722, 15.650 |
| Day 1 20 h Post-dose Effect (ms) | 6.75 | 2.2958 | 50.8 | 2.94 | 0.0049 | 2.903, 10.595 |
| Day 1 24 h Post-dose Effect (ms) | −0.090 | 2.3048 | 51.6 | -0.04 | 0.9689 | −3.9508, 3.7699 |
| Day 14 Pre-dose Effect (ms) | 1.89 | 2.2976 | 51.0 | 0.82 | 0.4136 | −1.955, 5.743 |
| Day 14 0.5 h Post-dose Effect (ms) | −0.51 | 2.3126 | 52.3 | -0.22 | 0.8276 | −4.379, 3.366 |
| Day 14 1 h Post-dose Effect (ms) | −0.26 | 2.3019 | 51.3 | -0.11 | 0.9121 | −4.111, 3.601 |
| Day 14 2 h Post-dose Effect (ms) | −3.30 | 2.3057 | 51.7 | -1.43 | 0.1584 | −7.161, 0.562 |
| Day 14 3 h Post-dose Effect (ms) | −3.97 | 2.3173 | 52.7 | -1.71 | 0.0923 | −7.853, −0.093 |
| Day 14 4 h Post-dose Effect (ms) | −1.72 | 2.3189 | 52.8 | -0.74 | 0.4606 | −5.606, 2.159 |
| Day 14 5 h Post-dose Effect (ms) | 2.18 | 2.3206 | 52.9 | 0.94 | 0.3510 | −1.702, 6.068 |
| Day 14 8 h Post-dose Effect (ms) | 1.22 | 2.3058 | 51.7 | 0.53 | 0.5999 | −2.645, 5.079 |
| Day 14 12 h Post-dose Effect (ms) | 2.49 | 2.3065 | 51.7 | 1.08 | 0.2849 | −1.371, 6.355 |
| Day 14 16 h Post-dose Effect (ms) | 3.58 | 2.4117 | 61.7 | 1.49 | 0.1425 | −0.444, 7.610 |
| Day 14 20 h Post-dose Effect (ms) | 6.67 | 2.3036 | 51.5 | 2.89 | 0.0056 | 2.809, 10.525 |
| Day 14 24 h Post-dose Effect (ms) | 1.32 | 2.3189 | 52.9 | 0.57 | 0.5722 | −2.564, 5.200 |

Based on a linear mixed-effects model with ΔQTcF as the dependent variable, time-matched M3 plasma concentration as an explanatory variable, centered baseline QTcF as an additional covariate, treatment (active = 1 or placebo = 0) and time as fixed effects, and a random intercept and random slope per subject. Fitted by REML estimation.
